# Penalized Maximum-Likelihood Reconstruction for Improving Limited-Angle Artifacts in a Dedicated Head and Neck PET System

**DOI:** 10.1088/1361-6560/ab8c92

**Published:** 2020-08-21

**Authors:** Hengquan Zhang, Yuli Wang, Jinyi Qi, Shiva Abbaszadeh

**Affiliations:** 1 Department of Nuclear, Plasma, and Radiological Engineering, University of Illinois at Urbana-Champaign, Champaign, IL, United States of America; 2 Department of Electrical and Computer Engineering, University of California - Santa Cruz, Santa Cruz, CA, United States of America; 3 Department of Biomedical Engineering, University of California - Davis, Davis, CA, United States of America

**Keywords:** Head and neck cancer, limited-angle artifacts, positron emission tomography, penalized maximum-likelihood image reconstruction

## Abstract

Positron emission tomography (PET) suffers from limited spatial resolution in current head and neck cancer management. We are building a dual-panel high-resolution PET system to aid the detection of tumor involvement in small lymph nodes (< 10 mm in diameter). The system is based on cadmium zinc telluride (CZT) detectors with cross-strip electrode readout (1 mm anode pitch and 5 mm cathode pitch). One challenge of the dual-panel system is that the limited angular coverage of the imaging volume leads to artifacts in reconstructed images, such as the elongation of lesions. In this work, we leverage a penalized maximum-likelihood (PML) reconstruction for the limited-angle PET system. The dissimilarity between the image to be reconstructed and a prior image from a low-resolution whole-body scanner is penalized. An image-based resolution model is incorporated into the regularization. Computer simulations were used to evaluate the performance of the method. Results demonstrate that the elongation of the 6-mm and 8-mm diameter hot spheres is eliminated with the regularization strength *γ* being 0.02 or larger. The PML reconstruction yields higher contrast recovery coefficient (CRC) of hot spheres compared to the maximum-likelihood reconstruction, as well as the low-resolution whole-body image, across all hot sphere sizes tested (3, 4, 6, and 8 mm). The method studied in this work provides a way to mitigate the limited-angle artifacts in the reconstruction from limited-angle PET data, making the high-resolution dual-panel dedicated head and neck PET system promising for head and neck cancer management.

## 1. Introduction

Head and neck cancer (HNC) arises in the oral cavity, nasal cavity, paranasal sinuses, pharynx, larynx, and salivary glands (Leemans, Braakhuis and Brakenhoff 2011, Vokes *et al* 1993, Escott 2013). Approximately 64,690 new cases of HNC are estimated to have occurred in the United States in 2017 (Cancer.Net 2017). Positron emission tomography (PET) has become a very useful tool in HNC management such as pre-treatment staging, radiation treatment planning, treatment response assessment and post-therapy followup (Escott 2013, Castaldi *et al* 2013, Özel 2015). Imaging in the head and neck is challenging due to the complex anatomy of this region and the small size of lymph nodes. Identifying nodal metastases is important, since even a single metastatic lymph node can significantly change the treatment and prognosis of a patient (Escott 2013). The limitation of PET is that it cannot detect microscopic disease due to the low spatial resolution of a typical whole body PET scanner (4–6 mm) (Castaldi *et al* 2013, Özel 2015, Yamamoto *et al* 2007). As a result, small lymph node lesions may be missed, causing false negative results. An improvement in spatial resolution of whole-body PET image (< 2 mm) to detect small lymph nodes (< 10 mm) and to better define the boundary of tumors will provide physicians with more freedom to choose treatment options, and more accurate radiation dose planning. For example, if supraglottic cell carcinoma involves thyroid cartilage, it is T4 and unresectable and if it does not involve thyroid cartilage, it is T3 and can be cured with surgery. State of the art clinical system that employ point spread function corrections can achieve ~3 mm spatial resolution (Lee *et al* 2014, van Sluis *et al* 2019).

**Figure 1.**
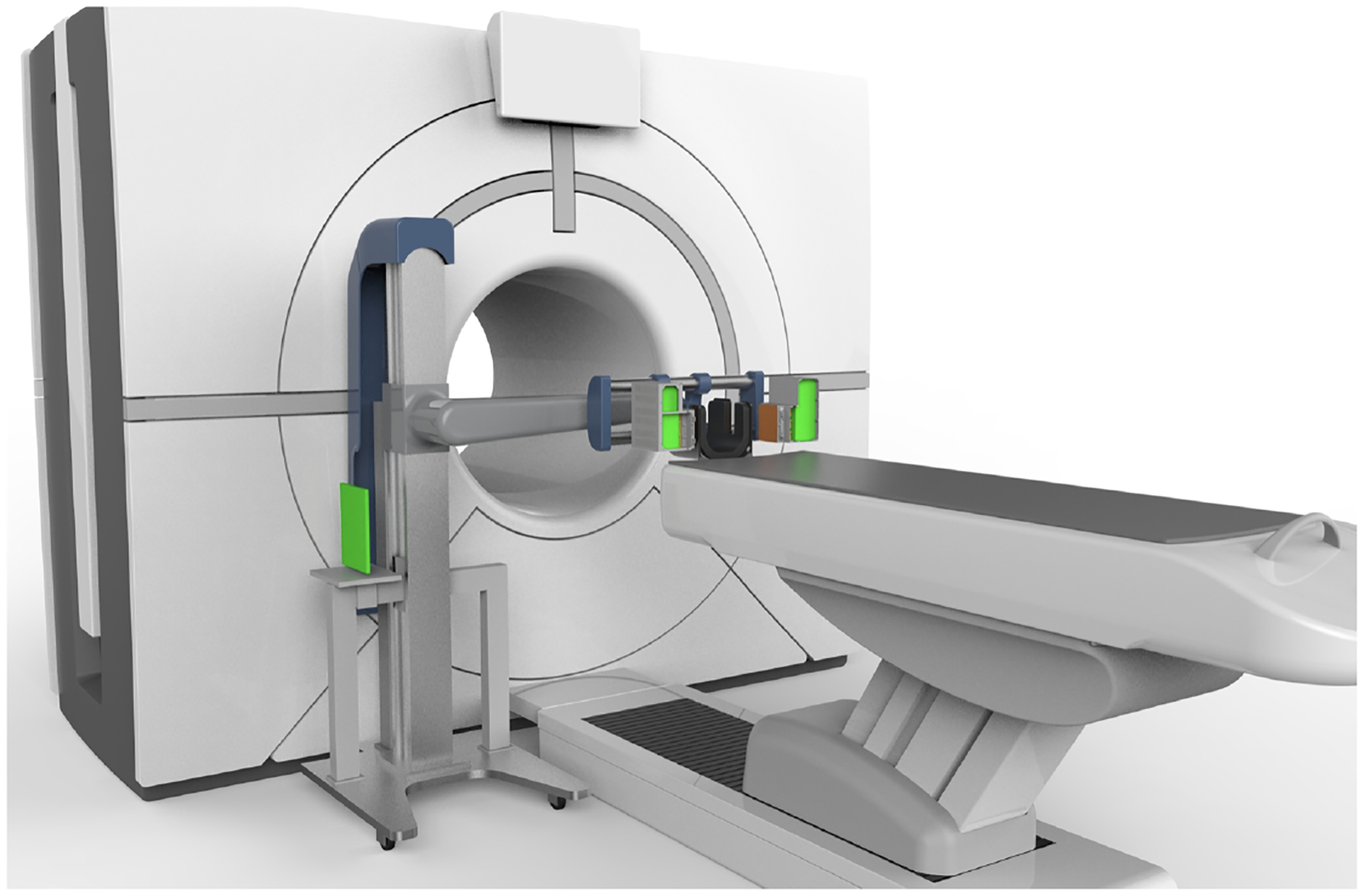
Illustration of the transportable dual-panel HNC dedicated PET system integrated into the imaging workflow.

We are developing a high-resolution PET system dedicated for head and neck imaging. The system is based on cadmium zinc telluride (CZT) detectors with cross-strip electrode readout. The cross-strip configuration requires fewer electronic readout channels for the same detector area than a fully pixelated anode (Li, Yockey and Abbaszadeh 2020). In the CZT detectors, detector pixelation is implemented electronically. The intersection of the anode and cathode strips that produce signal gives the position information of photon interactions. The intrinsic spatial resolution can be better than 1 mm with fine pitch of electrode strips, which cannot be easily achieved in scintillation detectors due to the complexity of cutting tiny crystals and assembling them into arrays (Abbaszadeh *et al* 2016). Compared to scintillation detectors, CZT detectors exhibit much better energy resolution for 511 keV photons but worse time resolution (Peng and Levin 2010). In addition, to accommodate patient comfort, a dual-panel design of the high-resolution PET system is employed, which minimizes the amount of system in the patient’s line of sight and prevents claustrophobia. The dual-panel system will be used after the patient undergoes the whole-body PET imaging. Figure 1 shows a conceptual impression of the transportable dual-panel head and neck gantry with an adjustable panel-to-panel separation distance. As a direct result from the clinical input, the gantry will be implemented to interface with the hospital PET bed to image the patient after the whole-body PET scan. The system panels will be adjusted to the already existing axial head holder. Thus, except for a 10–15 minute increase in total imaging time, there will be minimal impact on the conventional workflow. The dual-panel design has the flexibility for imaging organs and body parts that require high spatial resolution.

A challenge of the dual-panel system is that the limited angular coverage of the imaging volume produces artifacts in reconstructed images. The artifacts include image distortions in the direction perpendicular to the detector panels, such as the elongated shape of reconstructed spheres (Peng and Levin 2010, Surti and Karp 2008, Lee *et al* 2013, Matej *et al* 2016). One method of solving this problem is to rotate the detectors to obtain sufficient angular sampling, which leads to longer scan time and causes discomfort to patients. Several approaches to reducing limited-angle artifacts without detector rotation have been studied for breast-dedicated PET systems, such as time-of-flight (TOF) image reconstruction and image-based modeling of pointspread function (PSF) deformation (Surti and Karp 2008, Lee *et al* 2013, Matej *et al* 2016). In addition, researchers have proposed several methods of eliminating limited-angle artifacts for time-resolved computed tomography (CT) imaging where sufficient angular range for data acquisition is not achieved. These methods include prior image constrained compressed sensing (PICCS) and synchronized multi-artifact reduction with tomographic reconstruction (SMART-RECON) (Chen, Tang and Hsieh 2009, Chen and Li 2015). The limited-angle artifacts are eliminated by enforcing that the image to be reconstructed has similarity with a prior image of the same object (Chen, Tang and Hsieh 2009).

In this work, we study a method of reducing limited-angle artifacts for the proposed dual-panel PET system. The concept is similar to PICCS where a prior image reconstructed from the data acquired within a wide temporal window is used for constraining the image reconstruction from the data acquired in an ultra-narrow temporal window (Chen, Tang and Hsieh 2009). In this work, the prior image is pre-reconstructed from a whole-body PET scanner. A penalized maximum-likelihood (PML) image reconstruction is then performed with a regularization term that penalizes the dissimiliarity between the image to be reconstructed (the target image) and the prior image. An image-based resolution model is incorporated into the regularization term considering the limited spatial resolution of the whole-body PET scanner. The artifact mitigation is assessed both visually and quantitatively. The image quality is quantified by contrast recovery coefficient (CRC), background noise, and contrast-to-noise ratio (CNR).

## 2. Methods

### 2.1 Penalized Maximum-Likelihood Image Reconstruction

In PET image reconstruction, the likelihood function is expressed in the following form:

(1) $$P_{L}(y/x)=\prod_{i=1}^{I}{\bar{y}}_{i}^{y_{i}}\cdot \frac{e^{-{\bar{y}}_{i}}}{y_{i}!}$$

where

(2) $${\bar{y}}_{i}=\sum_{j=1}^{J}p_{ij}x_{j}$$

Here the detector measurements are modelled as a set of independent Poisson random variables *y*_*i*_(*i* = 1, 2, …, *I*) with mean ${\bar{y}}_{i}$ (Levitan and Herman 1987). *x*_*j*_ is the expected number of radioactive disintegrations from image voxel *j*; *p*_*ij*_ is the probability that an emission from voxel *j* will be detected by detector pair *i*; *I* and *J* are the number of detector pairs and voxels, respectively. Image $\hat{x}$ is estimated by maximizing the logarithm of the likelihood function *P*_*L*_(*y/x*):

(3) $$\hat{x}=\text{arg }\underset{x\in R_{+}^{J}}{\text{max}}\sum_{i=1}^{I}\left(y_{i}\text{ ln}\sum_{j=1}^{J}p_{ij}x_{j}-\sum_{j=1}^{J}p_{ij}x_{j}\right)$$

where $x\in R_{+}^{J}=\left\{x|x_{j}\geq 0,\text{ for }1\leq j\leq J\right\}$, (Herman, De Pierro and Gai 1992).

Our aim of implementing the PML image reconstruction with an image-based resolution model in the regularization is to eliminate the limited-angle artifacts in the target image while retaining the ability of the dedicated system to detect small lesions. The prior image does not have the limited-angle artifacts since it will be from a whole-body PET scanner. The artifacts in the target image reconstructed with the regularization will be reduced, since the dissimilarity between the blurred target image and the prior image is penalized. Let *γ* be a positive constant for controlling the regularization strength; *A* and *A*^*P*^ be an estimate of the average voxel values in the target image and the prior image, respectively. The regularization *F*(*x*) used for penalizing the dissimilarity between the prior image and the target image has the following form:

(4) $$F(x)=-\frac{\gamma \cdot A}{2}\sum_{j=1}^{J}{\left(\frac{b_{j}}{A}-\frac{x_{j}^{P}}{A^{P}}\right)}^{2}$$

where

(5) $$b_{j}=\sum_{k=1}^{J}w_{jk}x_{k}$$

Since the target image from the dual-panel scanner and the prior image from the whole-body scan have different spatial resolution, the target image is first blurred to the resolution of the prior image and scaled to the same intensity. Then the sum of the squared difference between the blurred target image and the whole-body prior image is taken. In equation (4), *b*_*j*_(*j* = 1, 2, …, *J*) is the blurred target image, $x_{j}^{P}$ is the prior image, and *γ* is a positive constant for controlling the regularization strength. The *γ* will determine how severe the penalization will be. The larger the penalization, the more similar the blurred target image will be to the prior image and vice versa. *w*_*jk*_ is the normalized weight in Gaussian blur, which is calculated as follows:

(6) $$w_{jk}=\frac{g_{jk}}{\sum_{k=1}^{J}g_{jk}}$$

where

(7) $$g_{jk}=\{\begin{array}{ll} \text{exp}\left(-\frac{d_{x}^{2}(j,k)}{2\sigma_{x}^{2}}-\frac{d_{y}^{2}(j,k)}{2\sigma_{y}^{2}}-\frac{d_{z}^{2}(j,k)}{2\sigma_{z}^{2}}\right), & \text{ if }g_{jk}>0.01\\ 0, & \text{ otherwise}, \end{array}$$

Here *d*_*a*_(*j*, *k*) (*a* = *x*, *y*, *z*) is the distance between the center of voxel *j* and the center of voxel *k* in the direction *a*; *σ*_*a*_ (*a* = *x*, *y*, *z*) will be calculated from the standard deviation of a Gaussian fit based on point source simulations for the whole-body scanner. The objective function that is maximized in the PML image reconstruction has the following form:

(8) G(x)=L(x)+F(x)

where *L*(*x*) is the log-likelihood function that is maximized in (3); *F*(*x*) is the regularization defined in (4).

The ML estimate of the target image can be computed iteratively using the expectation maximization (EM) algorithm (Levitan and Herman 1987, Lange and Carson 1984, Shepp and Vardi 1982). Let *z*_*ij*_ be the number of emissions from voxel *j* and detected by detector pair *i*. It has been shown (Levitan and Herman 1987, Lange and Carson 1984, Shepp and Vardi 1982) that the image at iteration *n* + 1 is estimated as follows:

(9) $$x^{\left(n+1\right)}=\text{arg }\underset{x\in R_{+}^{J}}{\text{max}}\left[E\left(\left(\text{ln }P_{L}(z/x)+F(x)\right)/y,x^{\left(n\right)}\right)\right]\;=\text{arg }\underset{x\in R_{+}^{J}}{\text{max}}\left[E\left(\text{ln }P_{L}(z/x)/y,x^{\left(n\right)}\right)+F(x)\right]\;=\text{arg }\underset{x\in R_{+}^{J}}{\text{max}}\left[\sum_{i=1}^{I}\sum_{j=1}^{J}\left(y_{i}\frac{p_{ij}x_{j}^{\left(n\right)}}{\sum_{m=1}^{J}p_{im}x_{m}^{\left(n\right)}}\text{ln }\left(p_{ij}x_{j}\right)-p_{ij}x_{j}\right)+F(x)\right]$$

The maximization step can be done by taking the first derivative of (9) with respect to *x*_*j*_ and equating it to zero. This will result in a set of coupled quadratic equations which are difficult to solve.

To obtain the iteration step for calculating *x*^(*n*+1)^, we utilized an algorithm proposed in (De Pierro 1995) that is a modification of the EM algorithm for penalized likelihood and is convergent for general concave penalizations. Let the regularization have the general form:

(10) $$F(x)=\sum_{l=1}^{p}f_{l}\left(\sum_{k=1}^{J}s_{lk}x_{k}-e_{l}\right)$$

where *f*_*l*_ (*l* = 1, 2, …, *p*) are strictly concave functions, twice continuously differentiable, and bounded (De Pierro 1995). For every fixed *l*, *s*_*lk*_ (*k* = 1, 2, …, *J*) are real-numbers at least one of which are nonzero. *e*_*l*_ (*l* = 1, 2, …, *p*) are real-numbers (De Pierro 1995).

The maximization step proposed in (De Pierro 1995) takes the following form:

(11) $$x^{\left(n+1\right)}=\text{arg }\underset{x\in R_{+}^{J}}{\text{max}}\left[E\left(\text{ln }P_{L}(z/x)/y,x^{\left(n\right)}\right)+Q_{2}\left(x,x^{\left(n\right)}\right)\right]\;=\text{arg }\underset{x\in R_{+}^{J}}{\text{max}}[\sum_{i=1}^{I}\sum_{j=1}^{J}\left(y_{i}\frac{p_{ij}x_{j}^{\left(n\right)}}{\sum_{m=1}^{J}p_{im}x_{m}^{\left(n\right)}}\text{ln }\left(p_{ij}x_{j}\right)-p_{ij}x_{j}\right)+\sum_{l=1}^{p}\sum_{k=1}^{J}\lambda_{k}^{l}f_{l}\left(c_{k}^{l}x_{k}+d_{k}^{ln}-e_{l}\right)]$$

Here, $\lambda_{k}^{l}\;(l=1,2,\ldots ,p;\;k=1,2,\ldots ,J)$ are nonnegative real numbers satisfying the conditions (De Pierro 1995) as follows:

(12) $$\lambda_{k}^{l}=0\text{ if and only if }s_{lk}=0,\;\sum_{k=1}^{J}\lambda_{k}^{l}=1\text{ for }l=1,2,\ldots ,p.$$

$c_{k}^{l}$ and $d_{k}^{ln}$ are defined (De Pierro 1995) as follows:

(13) $$c_{k}^{l}=\{\begin{array}{ll} \frac{s_{lk}}{\lambda_{k}^{l}}, & \text{ if }\lambda_{k}^{l}\neq 0\\ 0, & \text{ otherwise,} \end{array}\;d_{k}^{ln}=\sum_{m=1}^{J}s_{lm}x_{m}^{\left(n\right)}-c_{k}^{l}x_{k}^{\left(n\right)}.$$

For our problem *P* = *J*,*f*_*l*_(*x*) = −*γAx*^2^/2, *s*_*lk*_ = *w*_*lk*_/*A*, $s_{lk}=w_{lk}/A,\;e_{l}=x_{l}^{P}/A^{P}$. We chose $\lambda_{k}^{l}=w_{lk}$ which satisfies the conditions (12) according to the definition of *s*_*lk*_ in our problem (*s*_*lk*_ = *w*_*lk*_/*A*) and (6). By taking the first derivative of the objective function in (11) with respect to *x*_*j*_ and equating it to zero, we obtain the following quadratic equation for *x*_*j*_:

(14) $$\frac{x_{j}^{\left(n\right)}}{x_{j}}C_{j}^{\left(n\right)}-S_{j}+B_{j}^{\left(n\right)}=0,$$

where

(15) $$C_{j}^{\left(n\right)}=\sum_{i=1}^{I}\frac{y_{i}p_{ij}}{\sum_{m=1}^{J}p_{im}x_{m}^{\left(n\right)}},\;S_{j}=\sum_{i=1}^{I}p_{ij},\;B_{j}^{\left(n\right)}=-\gamma \sum_{l,\;w_{lj}\neq 0}w_{lj}\left(\left(x_{j}+b_{l}^{\left(n\right)}-x_{j}^{\left(n\right)}\right)/A-x_{j}^{P}/A^{P}\right).$$

By rearranging the terms in (14), we obtain

(16) $$a_{j}^{\left(n\right)}x_{j}^{2}+q_{j}^{\left(n\right)}x_{j}-v_{j}^{\left(n\right)}=0,$$

where

(17) $$a_{j}^{\left(n\right)}=\gamma \sum_{l,\;w_{lj}\neq 0}w_{lj}/A,\;q_{j}^{\left(n\right)}=S_{j}+\gamma \sum_{l,\;w_{lj}\neq 0}w_{lj}\left(\left(b_{l}^{\left(n\right)}-x_{j}^{\left(n\right)}\right)/A-x_{j}^{P}/A^{P}\right),\;v_{j}^{\left(n\right)}=x_{j}^{\left(n\right)}C_{j}^{\left(n\right)}.$$

The nonnegative solution of (16) is

(18) $$x_{j}^{\left(n+1\right)}=\left(-q_{j}^{\left(n\right)}+\sqrt{{\left(q_{j}^{\left(n\right)}\right)}^{2}+4a_{j}^{\left(n\right)}v_{j}^{\left(n\right)}}\right)/\left(2a_{j}^{\left(n\right)}\right).$$

Equation (18) is the unique solution of (11), since the objective function in (11) is strictly concave (Boyd and Vandenberghe 2004). It has been shown that for any *x*^(0)^ with positive components, the sequence generated by (18) converges to a global maximizer of (8) in $R_{+}^{J}$ (Levitan and Herman 1987, De Pierro 1995, Herman, De Pierro and Gai 1992).

For our proposed system, the number of possible lines of response (LOR) *I* is on the order of 10 billion which is much larger than the number of detected coincidence events *M*. List-mode image reconstruction (Huesman *et al* 2000, Parra and Barrett 1998) was utilized, resulting in a different method of calculating $C_{j}^{\left(n\right)}$:

(19) $$C_{j}^{\left(n\right)}=\sum_{v=1}^{M}\frac{p_{i_{vj}}}{\sum_{m=1}^{J}p_{i_{v}m}x_{m}^{\left(n\right)}}$$

where *i*_*v*_ is the detector pair index for the *v*th event.

### 2.2 Simulation Models

**Figure 2.**
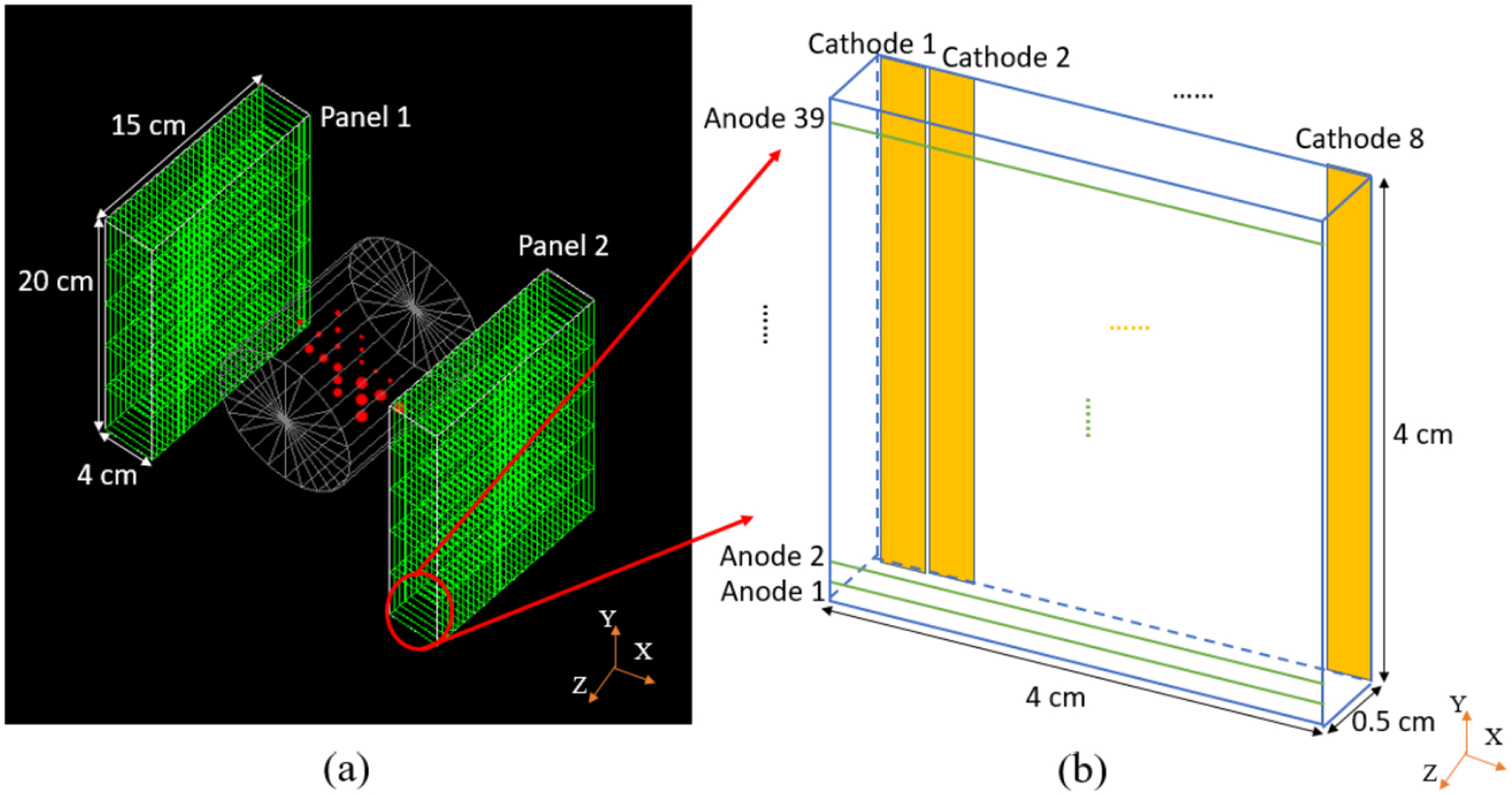
(a) Dual-panel dedicated head and neck PET scanner based on CZT detectors. Each panel consists of an array of 5 (Y) × 30 (Z) CZT detectors. (b) Schematic of a CZT detector. The anode pitch and cathode pitch are 1 mm and 5 mm, respectively. The intersection of the anode and cathode strips that produce signal gives the position information of photon interactions.

Monte Carlo simulations were performed in GATE (Geant4 Application for Tomographic Emission) (Jan *et al* 2004). The geometry of the proposed dual-panel dedicated head and neck PET scanner is as shown in Figure 2. The face-to-face distance between the two panels shown in Figure 2(a) is fixed at 20 cm in the simulations. The cross-strip CZT detector is as shown in Figure 2(b). The detectors are oriented edge-on with respect to incoming photons so that each incoming photon traverses up to 4 cm of CZT if it is perpendicular to the panel, or slightly more if it enters the panel at an angle. For the evaluation study, an 11-cm-diameter and 12.6-cm-long cylindrical phantom with uniform water attenuation was simulated. The phantom was approximately the same size as the neck of an adult (Yamamoto *et al* 2007). The background positron activity concentration was 5700 Bq/cm^3^ in the simulations. Considering the F-18 branching ratio for *β*^+^ decay is 97% (Jacobson, Kiesewetter and Chen 2015), the equivalent concentration is 5893 Bq/cm^3^ in ^18^F-FDG (fluorodeoxyglucose), similar to the activity during a typical acquisition (Yamamoto *et al* 2007, Mathews *et al* 2013). Hot spheres were put inside the phantom at the center slice, including a set of five 3-mm diameter spheres, five 4-mm diameter spheres, five 6-mm diameter spheres, and five 8-mm diameter spheres, as shown in Figure 2(a). The distance between two neighboring spheres was at least twice the diameter of the spheres. The sphere to background ratio was 8:1, which is consistent with FDG tracer uptake in head and neck cancer (Lindholm *et al* 1993). The simulated acquisition time was 10 minutes with 30 million coincidence events acquired.

A coincidence sorting method was developed to generate coincidence events from ‘hits’ data. The deposited energy and time stamp were blurred according to Gaussian distribution based on the experimentally measured detector energy resolution (3% full-width at half maximum (FWHM) at 511 keV) and time resolution (18 ns FWHM) (Gu *et al* 2011). The FWHM energy resolution, *R*_*E*_, is calculated as (Jan *et al* 2004):

(20) $$R_{E}=R_{511}\sqrt{\frac{511}{E}}$$

where *R*_*E*_ and *R*511 are the energy resolutions at *E* and 511 keV, respectively. The position of a photon interaction was binned to the center of the nearest 3D detector voxel with size being 5 mm (X) × 1 mm (Y) × 1 mm (Z). The spatial resolution in X and Y direction depends on anode pitch and cathode pitch. The position along the direction orthogonal to the electrode planes of the CZT detector (coordinate in Z direction) can be measured using cathode-to-anode signal ratio (C/A ratio), with resolution higher than 1 mm achievable (Gu *et al* 2011). For an event involving more than one interaction, the first interaction point was identified using the Compton kinematics algorithm developed in (Abbaszadeh, Chinn and Levin 2018) for both inter-crystal and intra-crystal multiple interactions. A coincidence time window of 25 ns and an energy window of ±6% around the photopeak (480 keV - 542 keV) were used in coincidence sorting, which had been optimized to achieve highest noise equivalent count rate (NECR) (Peng and Levin 2010) with the cylindrical phantom.

The prior image used in PML image reconstruction was from a whole-body scanner. The geometry of the whole-body scanner used in simulations was referred from the Discovery MI 4-ring PET scanner (GE Healthcare) (Hsu *et al* 2017, Grant *et al* 2016). The GATE digitizer was utilized for coincidence sorting. The digitizer setting was based on the performance specifications for the PET system (Grant *et al* 2016). In addition, the coordinate outputs for a given coincidence event were rebinned to the center of the nearest scintillation crystal. The size of an individual crystal was 25 mm (length) × 4.0 mm (transaxial) × 5.3 mm (axial) (Grant *et al* 2016). The simulation time was 10 minutes with 81 million coincidence events acquired.

For obtaining the parameters (*σ*_*x*_, *σ*_*y*_, *σ*_*z*_) of the resolution model in the regularization, we simulated a set of point sources in the cylindrical phantom located at 10-mm increments along the the radial direction for the whole-body scanner. The background was set to have a low level of activity to represent the simulated use case while still being able to resolve the point sources. The number of iterations used was the same as for generating the prior image (60). 3D Gaussian fit was done to each reconstructed point source. We took averages of the widths from the Gaussian fit, *σ* = 1.7 mm, consistent with previously reported values (Hsu *et al* 2017), and used them as the widths in the resolution model. Although the resolution kernel should model the difference between the two systems, in our application the high-resolution system contributes to a negligible amount, and is not accounted for in this work.

### 2.3. Image Reconstruction and Analysis

Reconstructions were performed using the orthogonal distance-based ray-tracer (ODRT) as the geometrical projector (Aguiar *et al* 2010). A fixed-width Gaussian kernel centered on the LOR axis was utilized to represent the system response (Aguiar *et al* 2010, Pratx *et al* 2009). The voxel size in the reconstructed images for the dual-panel scanner and the whole-body scanner were 0.5 mm and 2 mm, respectively. A trilinear interpolation method was used to interpolate the image from the whole-body scanner to obtain the prior image at the same voxel size as the target image. Fully 3D list-mode image reconstructions were implemented using the compute unified device architecture (CUDA) framework on a local workstation equipped with one NVIDIA P6000 (NVIDIA Corporation) graphics processing unit (GPU) card. Data corrections for scatter and random coincidence, which accounted for ~8% and ~8.7% of the total coincidences respectively, were not applied. For the normalization image we simulated a uniform cubic source that emitted back-to-back 511 keV gamma-rays. The source occupied the entire field of view (FOV), which had the size of 110 mm (X) × 110 mm (Y) × 130 mm (Z). The attenuation was modelled using a water phantom which had the same shape and size as the phantom used for the evaluation study. We simulated 80 million coincidence events for the dual-panel system and 100 million events for the whole-body system.

Limited-angle artifacts were assessed both visually and quantitatively. For quantitative analysis, we used the normalized root mean square deviation (NRMSD) metric (Chen and Li 2015, Gaitanis *et al* 2010). The NRMSD is defined as:

(21) $$\text{NRMSD}=\sqrt{\frac{\sum_{j=1}^{J}{\left(x_{j}-x_{j}^{\text{truth}}\right)}^{2}}{\sum_{j=1}^{J}{\left(x_{j}^{\text{truth}}\right)}^{2}}},$$

where *x*_*j*_ and $x_{j}^{\text{truth}}$ are the image being assessed and the ground truth image, which have been scaled by the average counts in the entire image.

**Figure 3.**
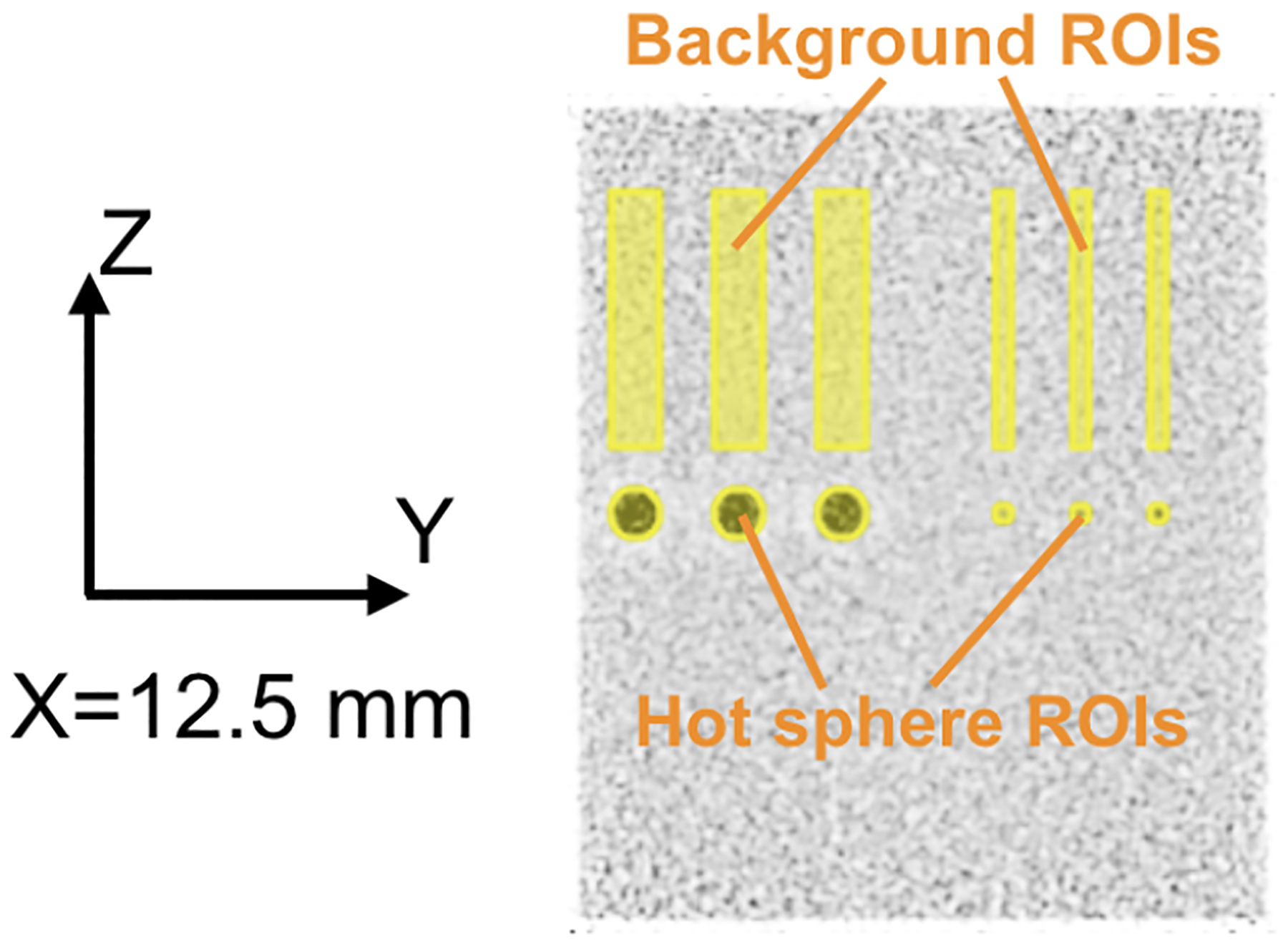
Sagittal slice (X = 12.5 mm) of the reconstructed image for the cylindrical phantom with hot spheres scanned by the dual-panel dedicated head and neck PET scanner. To calculate CRC and background noise, hot sphere ROIs and background ROIs are drawn. A hot sphere ROI has the same position and size as the corresponding hot sphere. The background ROIs are cylinders. A background ROI has the same diameter as the corresponding hot sphere ROI. The center of a background ROI has the same trans-axial coordinates (X and Y) as the center of the corresponding hot sphere ROI.

For image quality analysis, we used CRC and background noise (Abbaszadeh, Chinn and Levin 2018, Surti and Karp 2008). CRC was calculated as:

(22) $$CRC=\frac{C_{hot}/C_{bkg}-1}{A_{hot}/A_{bkg}-1}$$

where *C*_*hot*_ and *C*_*bkg*_ are the average counts in a hot sphere region of interest (ROI) and a background ROI, respectively; *A*_*hot*_ and *A*_*bkg*_ are the activity concentration in hot sphere and background, respectively. Background noise was calculated as:

(23) $$N=\frac{\sigma_{bkg}}{C_{bkg}}$$

where *σ*_*bkg*_ is the standard deviation of the counts in the background ROI. The hot sphere ROI and background ROI are as shown in Figure 3.

## 3. Results

### 3.1. Limited-Angle Artifacts

**Figure 4.**
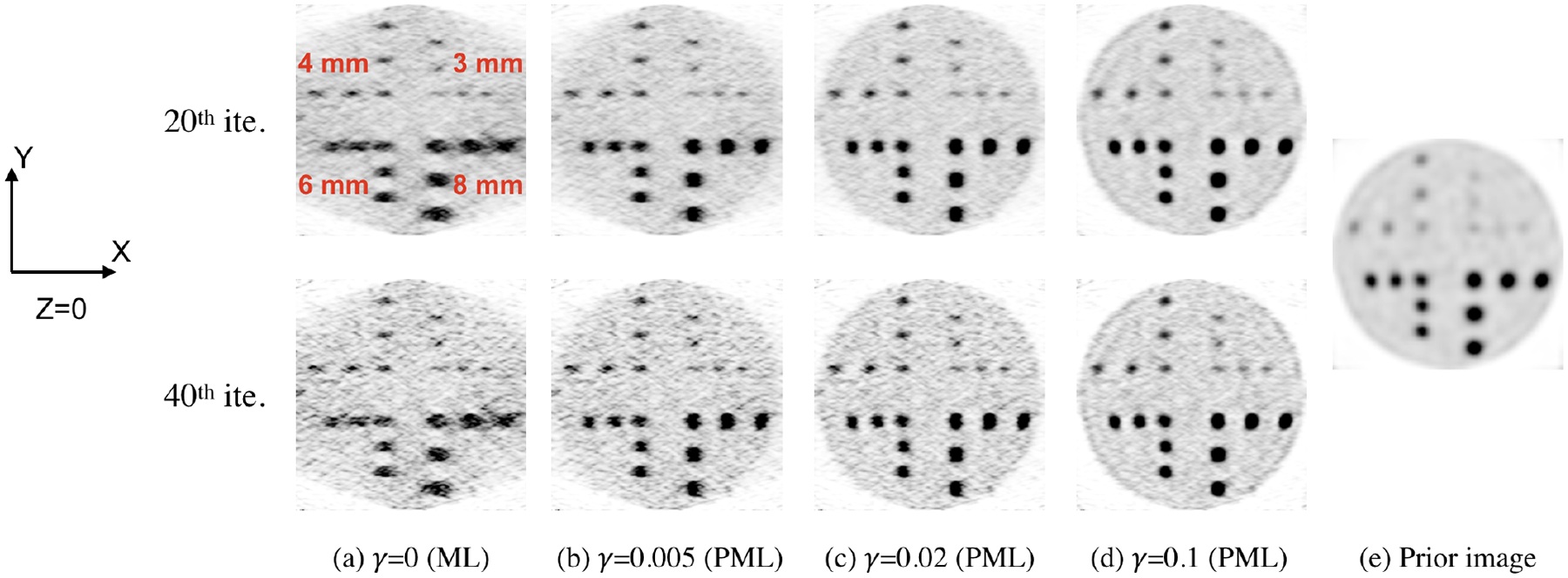
Transverse slices (Z = 0) from reconstructed images for the cylindrical phantom with hot spheres. The images in (a) – (d) are transverse slices from target images reconstructed from the dual-panel high-spatial-resolution PET scan of the phantom. The images in (a) are from the ML reconstruction without any regularization. The images in (b) – (d) are from the penalized maximum-likelihood (PML) reconstruction. The image in (e) is a transverse slice from the prior image reconstructed from the whole-body PET scan of the phantom, which is used for regularizing the image reconstruction for the dual-panel scanner. Each image has 220 × 220 pixels, with the size of each pixel being 0.5 mm × 0.5 mm.

**Figure 5.**
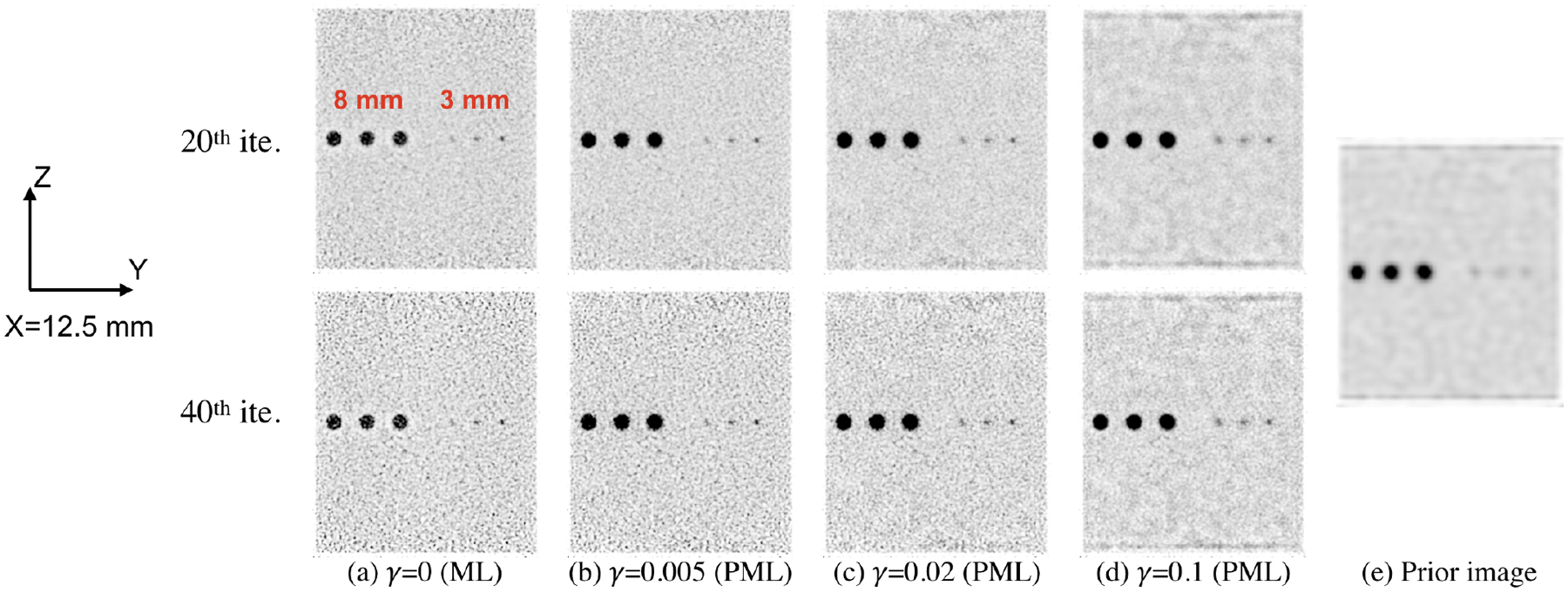
Sagittal slices (X = 12.5 mm) through 3-mm and 8-mm diameter hot spheres from reconstructed images for the cylindrical phantom with hot spheres. The images in (a) – (d) are sagittal slices from target images reconstructed from the dual-panel high-spatial-resolution PET scan of the phantom. The images in (a) are from the ML reconstruction without any regularization. The images in (b) – (d) are from the penalized maximum-likelihood (PML) reconstruction. The image in (e) is a sagittal slice from the prior image reconstructed from the whole-body PET scan of the phantom, which is used for regularizing the image reconstruction for the dual-panel scanner. Each image has 220 (Y) × 260 (Z) pixels, with the size of each pixel being 0.5 mm × 0.5 mm.

**Figure 6.**
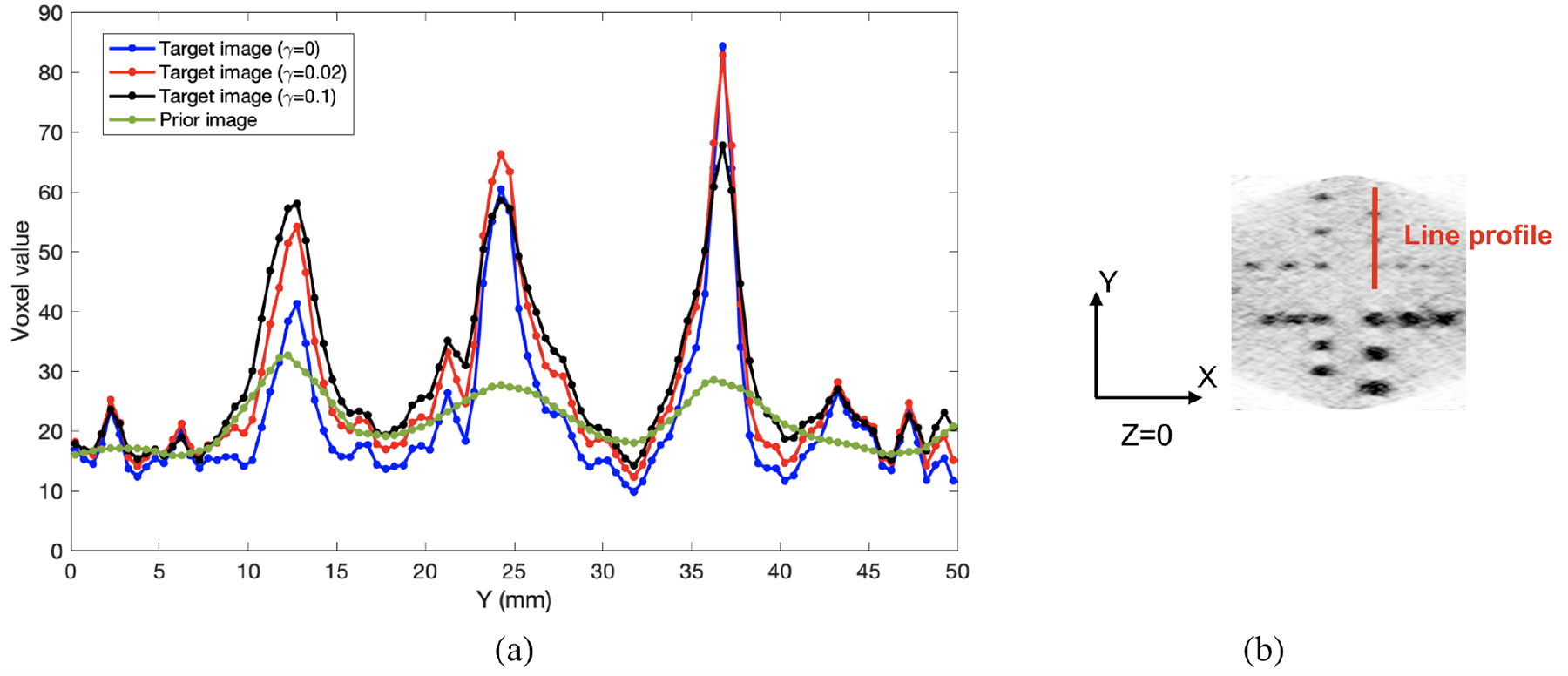
(a) Line profiles through three 3-mm diameter hot spheres at the 20^th^ iteration for different target images and the prior image. The Y coordinates of the three spheres are 12.5 mm, 24.5 mm, and 36.5 mm. (b) Transverse slice (Z = 0) from the target image reconstructed from the dual-panel high-spatial-resolution PET scan of the cylindrical phantom at 20^th^ iteration with being 0. The vertical line indicates the location of the line profiles in (a).

The transverse slices and sagittal slices from reconstructed images for the cylindrical phantom with hot spheres (as shown in Figure 2(a)) are shown in Figure 4 and Figure 5, respectively. The images at 20th and 40th iterations are both shown in the figures. The ML reconstruction has strong limited-angle artifacts. The background and hot spheres are both elongated, as shown in Figure 4(a). With PML image reconstruction, the artifacts are mitigated, as shown in Figure 4(b)–(d). The PML reconstructions were computed for three different values of the regularization strength *γ* (0.005, 0.02, and 0.1). For the reconstruction with *γ* being 0.005, the elongation of the 8-mm and 6-mm diameter hot spheres is eliminated, whereas the elongation of the uniform background is still noticeable. As *γ* increases from 0.005 to 0.02, the reconstructed image has the disk-shaped background, which is consistent with the ground truth. The limited-angle artifacts are noticeable for the 4-mm and 3-mm diameter hot spheres with *γ* being 0.005 or 0.02. The three 3-mm diameter hot spheres with the same X coordinate are challenging to resolve in the prior image, and they are resolvable in the target image reconstructed with *γ* being 0, 0.005, 0.02, or 0.1, as shown in Figure 4 and Figure 5. The line profiles through the three 3-mm diameter hot spheres are shown in Figure 6. The line profiles of the target images reconstructed with the various *γ* clearly show three peaks corresponding to the three hot spheres. The peaks are not as resolvable in the line profile of the prior image, and had the spheres been closer to each other than what was simulated here, it is likely they would not have been resolvable.

**Figure 7.**
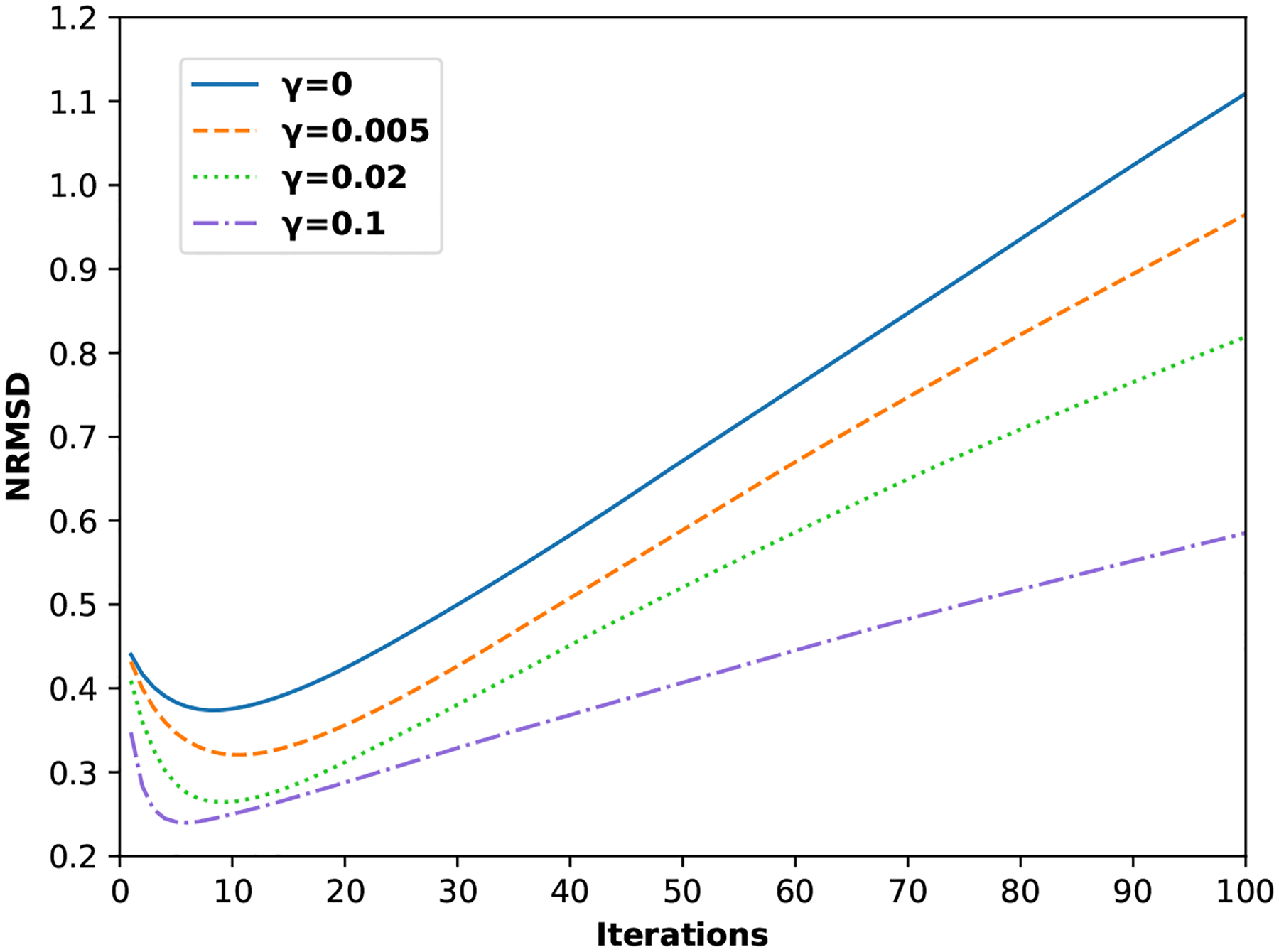
NRMSD as a function of the number of iterations in image reconstruction for different regularization strength. The larger the NRMSD, the larger the degree of deviation of the reconstructed image from the truth.

The NRMSD curves with four different values of *γ* are shown in Figure 7. For the same *γ*, NRMSD first decreases and then increases as the number of iterations increases. NRMSD measures the degree of deviation of the reconstructed image from the ground truth. At the beginning, the image becomes more similar to the ground truth after each iteration. Then, the image starts to deviate from the ground truth at higher iterations. The reason is that the noise level in the image increases as the number of iterations increases, which is known as the ”checkerboard effect” (Levitan and Herman 1987) in ML image reconstruction. The trend of the NRMSD curve is similar for both the ML and PML reconstruction. As *γ* increases, the curve achieves lower values at the same iteration, which indicates that the reconstructed image with larger *γ* is more similar to the ground truth.

**Figure 8.**
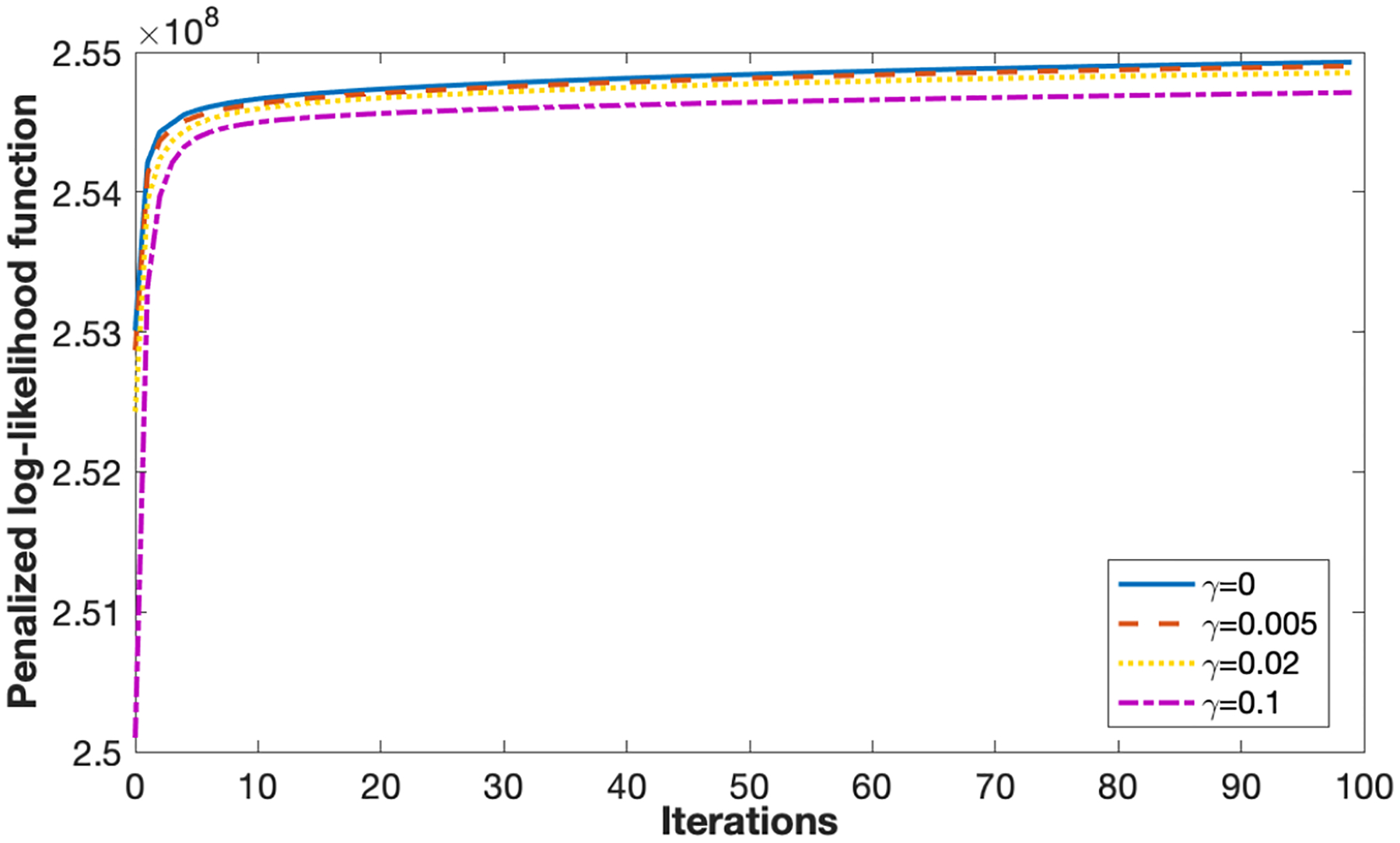
Penalized log-likelihood function (PLF) curve obtained for different regularization strength. As the number of iterations increase, PLF increases rapidly at first and then plateaus.

The penalized log-likelihood function (PLF) curves with four different values of *γ* are shown in Figure 8. As the number of iterations increases, the PLF first increases rapidly then gradually plateaus. This is expected as the PLF is the objective function which is maximized in the image reconstruction. The PLF converges to a lower value as *γ* increases, which is due to the increasing weight of the regularization term.

### 3.2. Image Quality

**Figure 9.**
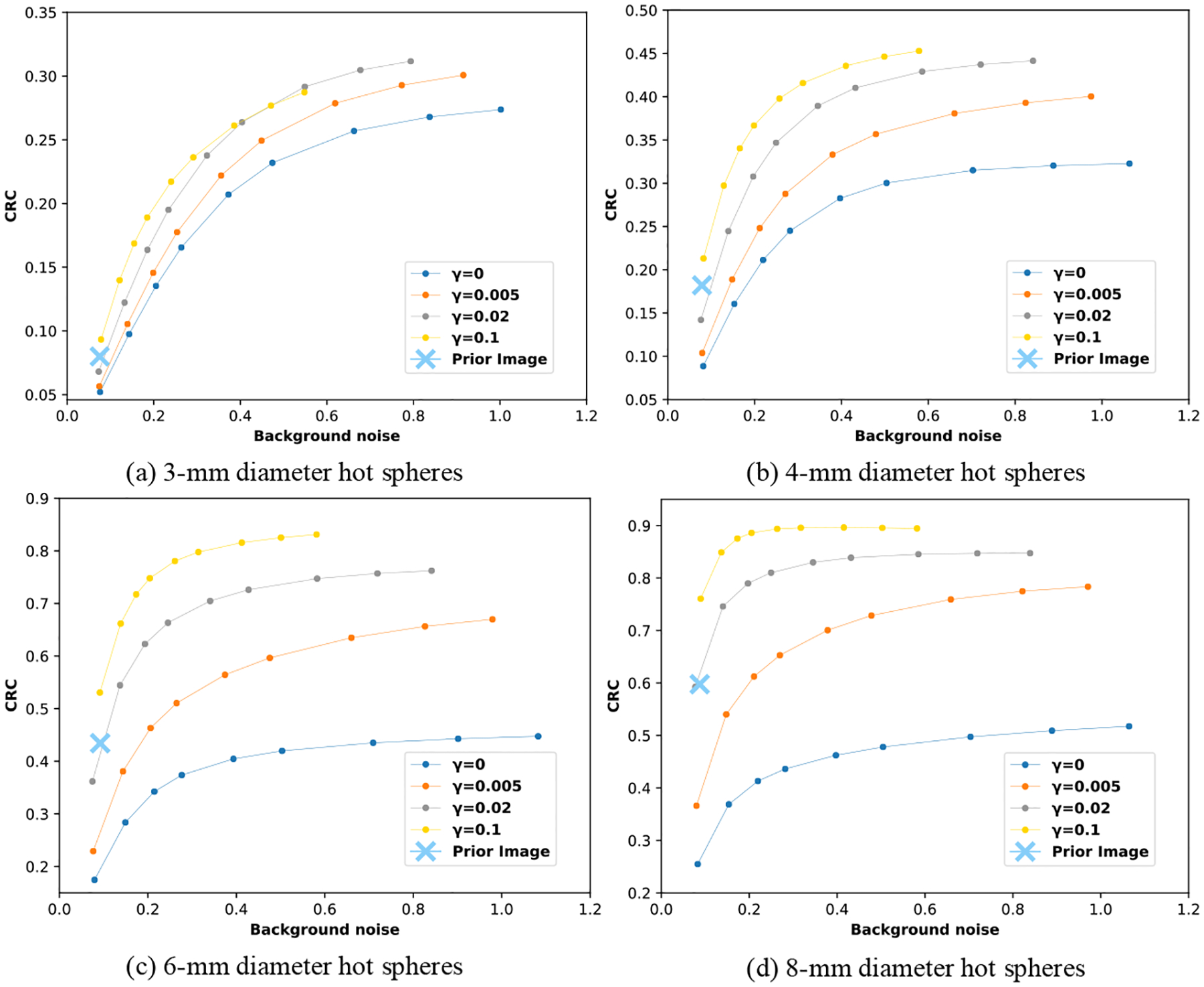
The contrast recovery coefficient (CRC) of hot spheres versus background noise with different regularization strength *γ*. The plots are based on a 10 minute scan of the cylindrical phantom with hot spheres by the proposed dual-panel dedicated head and neck PET scanner. The prior image is from the whole-body scanner used for regularizing the image reconstruction for the dual-panel scanner. Different plots correspond to spheres of different size. The data points in each curve, from left to right, correspond to iteration 5, 10, 15, 20, 30, 40, 60, 80, and 100, respectively. In general, as the number of iterations increases, background noise and CRC both increase.

The CRC curves versus background noise curves are shown in Figure 9. In each curve, different data points represent the images at different iterations. In general, as the number of iterations increases, the background noise and the CRC of the spheres increase. The CRC increases rapidly at first and then converges while the background noise keeps increasing. Figure 9 shows that the CRC converges at lower noise level for the image reconstructed with larger *γ*.

The CRC converges, in general, to a higher value as *γ* increases, and the difference becomes less pronounced between *γ* of 0.02 and 0.1. For the background noise levels shown, it is possible to achieve higher CRC using the PML reconstruction than using the ML reconstruction. Although regularization improves CRC in all sphere sizes, the improvement in CRC offered by PML is greatest for the largest sphere size, where the limited-angle artifacts appeared to be the most evident (Figure 5).

The CRC and the background noise for the prior image reconstructed from the whole-body scan is also shown in Figure 9. The prior image has higher CRC of the 8-mm diameter hot spheres than the target image reconstructed without any regularization (*γ* = 0). Similar to case mentioned above, the reason is that the 8-mm diameter hot spheres are more blurred in the X-direction in the target image reconstructed without regularization (11.3 mm FWHM from Gaussian fit) than in the prior image (5.93 mm FWHM from Gaussian fit) due to the limited-angle geometry of the dual-panel scanner. This is why the PML reconstruction improves the contrast of 8-mm spheres over the ML reconstruction. The CRC of the spheres in the target image reconstructed with PML is higher than the CRC in the prior image. The resolution model incorporated into the regularization allows the target image to maintain high contrast while correcting for limited-angle artifacts. As the regularization strength increases, the blurred target image, and as a consequence the target image as well, becomes more similar to the prior image, as shown in Figure 4 (d) and (e). If *γ* is infinite, the blurred target image instead of the target image itself is the same as the prior image.

## 4. Discussion

In this work, we have presented a penalized maximum-likelihood image reconstruction method for reducing limited-angle artifacts in the reconstruction for a dual-panel dedicated head and neck PET system based on CZT detectors. Due to the limited angular coverage of the imaging plane, ML reconstruction will have strong limited-angle artifacts. The PML reconstruction method that we proposed in this work incorporates a regularization term into the objective function in the ML estimation of the target image, which penalizes the dissimilarity between the blurred target image and a prior image without limited-angle artifacts. The prior image will be from a whole-body scanner, since the dedicated PET system will be used right after standard PET/CT, using the same bed and FDG dose. Our reconstructions of a cylindrical phantom with hot spheres show that the elongation of the warm background region, and the hot spheres is eliminated when the regularization strength *γ* is at least 0.02. The NRMSD versus number of iteration curves in Figure 7 show that the minimum NRMSDs correspond to the reconstructions with *γ* being 0, 0.005, 0.02, and 0.1 are 0.37, 0.32, 0.26, and 0.24, respectively. The results indicate that the images reconstructed with regularization are closer to the ground truth than the image reconstructed without regularization. For the four different values of *γ* studied in this work, the NRMSD curves reach a minimum at around 10^th^ iteration. The decrease in NRMSD is mainly due to the mitigation of limited-angle artifacts, not noise, since the noise level is almost the same in the four reconstructions at iteration 10, according to Figure 9. Since the NRMSD is not sensitive to local reconstruction features such as the contrast of small hot spheres, the image quality related to lesion detection is studied through CRC instead.

We have investigated the influence of the regularization strength *γ* on the CRC of hot spheres. The prior image reconstructed from the whole-body scan has higher CRC than the target image reconstructed without regularization for the case of the 8-mm diameter hot spheres, where the limited-angle artifact was most pronounced. Since the regularization forces the blurred target image to be similar to the whole-body image, the CRC of the spheres is increased in the reconstructions with regularization compared to the reconstruction without any regularization. The resulting CRC for the reconstructions with regularization is also significantly higher than that of the prior image.

The dual-panel high-spatial-resolution PET scanner is better at imaging small tumors than the whole-body scanner. Nevertheless, the limited-angle artifacts make the shape of reconstructed objects deviate from ground truth. By using the PML reconstruction method with the whole-body image of the same object as constraint, the limited-angle artifacts are almost eliminated. Compared to the whole-body image, the PML reconstruction with appropriate regularization strength *γ* achieves higher CRC of hot spheres and can resolve small features like the 3-mm and 4-mm diameter hot spheres, as shown in Figure 4, Figure 5, Figure 6, and Figure 9.

It should be noted that in this paper, due to the small fraction of scatter and random events in the simulated phantom, scatter and random correction was not implemented. However, in a clinical application this will not be the case. For example, there will be more random events due to activity outside the FOV of the dual-panel system, such as from the torso. Additionally, since the PML reconstruction method involves the whole-body PET scan and the dual-panel dedicated head and neck PET scan, patient motion may occur between the two scans, making the whole-body data and the dual-panel data misaligned. This problem can be solved by realigning the reconstructed whole-body prior image using patient’s motion information (e.g. translation and rotation) before PML reconstruction and is part of ongoing work. The patient’s motion information can be obtained by utilizing a motion tracking system (error less than 1 mm achievable) (Rahmim, Rousset and Zaidi 2007, Lopresti *et al* 1999, Fulton *et al* 2002).

## 5. Conclusions

We have shown that the penalized maximum-likelihood image reconstruction method can mitigate the limited-angle artifacts for the dual-panel dedicated head and neck PET system. The elongation of the large features (background, 6-mm and 8-mm diameter hot spheres) is eliminated with a proper choice of the regularization strength (*γ* ≥ 0.02). With a proper choice of regularization strength and reaching convergence, the ability for the system to resolve the small features (3-mm diameter hot sphere that cannot be easily resolved by the whole-body system) is retained in Y and Z directions. The method studied in this work will make the dual-panel CZT-based PET system promising for translating high spatial resolution detector technology to head and neck cancer management.
